# PCR-diagnosis of *Anaplasma marginale* in cattle populations of Ecuador and its molecular identification through sequencing of ribosomal 16S fragments

**DOI:** 10.1186/s12917-017-1311-1

**Published:** 2017-12-15

**Authors:** Leandro Tana-Hernández, Katherine Navarrete-Arroyo, Jorge Ron-Román, Armando Reyna-Bello, María Augusta Chávez-Larrea

**Affiliations:** 1 Grupo de Investigación en Sanidad Animal y Humana (GISAH), Carrera Ingeniería en Biotecnología, Departamento de Ciencias de la Vida y la Agricultura, Universidad de las Fuerzas Armadas ESPE, Sangolquí, Ecuador; 2 Grupo de Investigación en Sanidad Animal y Humana (GISAH), Carrera Ingeniería Agropecuaria, Departamento de Ciencias de la Vida y la Agricultura, Universidad de las Fuerzas Armadas ESPE, Sangolquí, Ecuador; 3 Investigador-Prometeo, Departamento de Ciencias de la Vida, Carrera Ingeniería en Biotecnología, Universidad de las Fuerzas Armadas ESPE, Sangolquí, Ecuador; 4 Carrera de Ingeniería en Biotecnología, Departamento de Ciencias de la Vida y la Agricultura, Universidad de las Fuerzas Armadas – ESPE, Av. General Rumiñahui s/n, P.O.BOX: 171-5-231, Sangolquí, Ecuador

**Keywords:** Bovine Anaplasmosis, *Anaplasma marginale*, *msp5*, 16S rRNA, Bovine disease, Ecuador

## Abstract

**Background:**

Bovine anaplasmosis is an endemic disease in tropical and subtropical areas. It is caused by a bacterium named *Anaplasma marginale*, and represents an economic problem for cattle farmers due to the losses it generates, such as: mortalities, reduced production, quarantine measures, treatments and control of vectors. The method most often used to diagnose this haemotrophic bacterium is direct examination on blood smear, which sensitivity and specificity are limited compared to other methods such as PCR. The present study aimed at investigating the presence of *A. marginale* in dairy cattle of Luz de América commune, province of Santo Domingo de los Tsachilas. Two PCRs were used to amplify specific regions of the *Rickettsia* for its molecular identification.

**Results:**

At first, 151 blood samples were tested: *msp*5 specific gene of *A. marginale* was identified in 130 samples, meaning 86.1% of them were infected by the rickettsia*.* Two positive samples were further randomly selected to confirm the presence of *A. marginale* through amplification, cloning and sequencing of the conserved region of gene 16S rRNA. The analysis of sequences obtained through cloning revealed a 100% identity between both samples and those registered in GenBank for *A. marginale*.

**Conclusion:**

This is the first report and molecular identification of *A. marginale* in the bovine population of Ecuador and its prevalence was high at the level of farms and animals. These results demonstrate the importance of proceeding to evaluate and characterize bovine Anaplasmosis in Ecuador in order to establish control measures and reduce their impact.

## Background

Bovine anaplasmosis is a vector-borne disease caused by the rickettsia *Anaplasma marginale* (*A. marginale)*. The disease is mainly characterized by fever, anaemia, weight loss, pale mucous membranes and sometimes death of affected animals. Its distribution includes the whole American continent, Asia, Africa, Europa and Australia, and generates reduced production, increased costs and hinders genetic improvement through the difficulty of introducing susceptible animals in endemic herds [1–3].

The distribution of the bacteria depends on the presence/absence of vectors, which are arthropods belonging to the Family Ixodidae; the most important are genera *Dermacentor* and *Rhipicephalus*. In Latin America, the tick of major distribution is *Rhipicephalus microplus* (*R. microplus*), which is incriminated as vector of anaplasmosis [4]. However, the epidemiological importance of ticks in the eco-epidemiology of the disease in Latin America is controversial, as the transmission of the rickettsia by blood sucking insects, such as horseflies, would be more important [4, 5].

Most animals positive for anaplasmosis are permanently infected, with a rickettsemia ranging from 10^4^ to 10^7^ [6]. They are responsible for an epidemiological status known as ‘enzootic stability’ in herds [4]. As the majority of animals in this condition have a low rickettsemia and do not show clinical signs, diagnosis is difficult [1, 6, 7].

Serological and molecular diagnoses are the only methods allowing the detection of *A. marginale*, as their sensitivity and specificity are high [8]. Polymerase Chain Reaction (PCR), based on amplification of DNA fragment, has been recommended to detect infection in animals to be commercialized and/or moved internationally [9]. A positive result to the Enzyme Linked Immunosorbent Assay (ELISA) only confirms the presence of the pathogen at some time, as it detects antibodies; it does not necessarily mean the pathogen is present by the time the test is performed [8]. Culture and isolation of the causing agent are frequently used as gold standard methods for diagnosing other bacterial diseases; they are not applicable in the case of *A. marginale* as it cannot be cultured [10]. The unique gold standard remains xenodiagnosis which is not very convenient [8, 9]. For such reason, PCR recommended as confirmatory test for the diagnosis of bovine anaplasmosis [11]. In addition to PCR, the sequencing of 16S of ribosomal RNA (16S rRNA**)** gene allows identifying genus and species of these microorganisms; it has thus been used concomitantly with groESL operon for further classification [10].

Despite the tropical distribution of bovine anaplasmosis, little has been done to confirm the presence of the disease in Ecuadorian cattle, even though horseflies and *R. microplus* ticks are present in Ecuador [12, 13]. However, neither spatiotemporal distribution, risk factors associated with the disease nor characterization of the causing agent has been studied deeply in the country. The scarce scientific information available is mainly found in theses performed in universities and in non-indexed journals. For example a 68%-prevalence was recently reported by Muñoz and collaborators after using blood smear [14]. On the other hand, a prevalence of 85.5% was estimated by nested PCR in cattle sampled in the province of Los Rios, Quevedo canton [15], while Soto reported a prevalence of 91.7% by using a commercial ELISA [16].

The rickettsia was even identified through sequencing of 16S rRNA gene in *R. microplus* ticks collected on two cows [13]. In view of these preliminary results, one could think the disease is endemic in cattle of Ecuador. In order to collect a better information on the presence of bovine anaplasmosis, a PCR based on the detection of the DNA fragment 605 bp of Mayor Surface Protein 5 (*msp5*) [17] was standardized and tested in animals located in an area where vectors are present. Subsequently, a sequencing of previously cloned 16S rRNA obtained from two samples collected at random was performed for confirmation.

## Methods

### Type of study and geographic localization

**Fig. 1:**
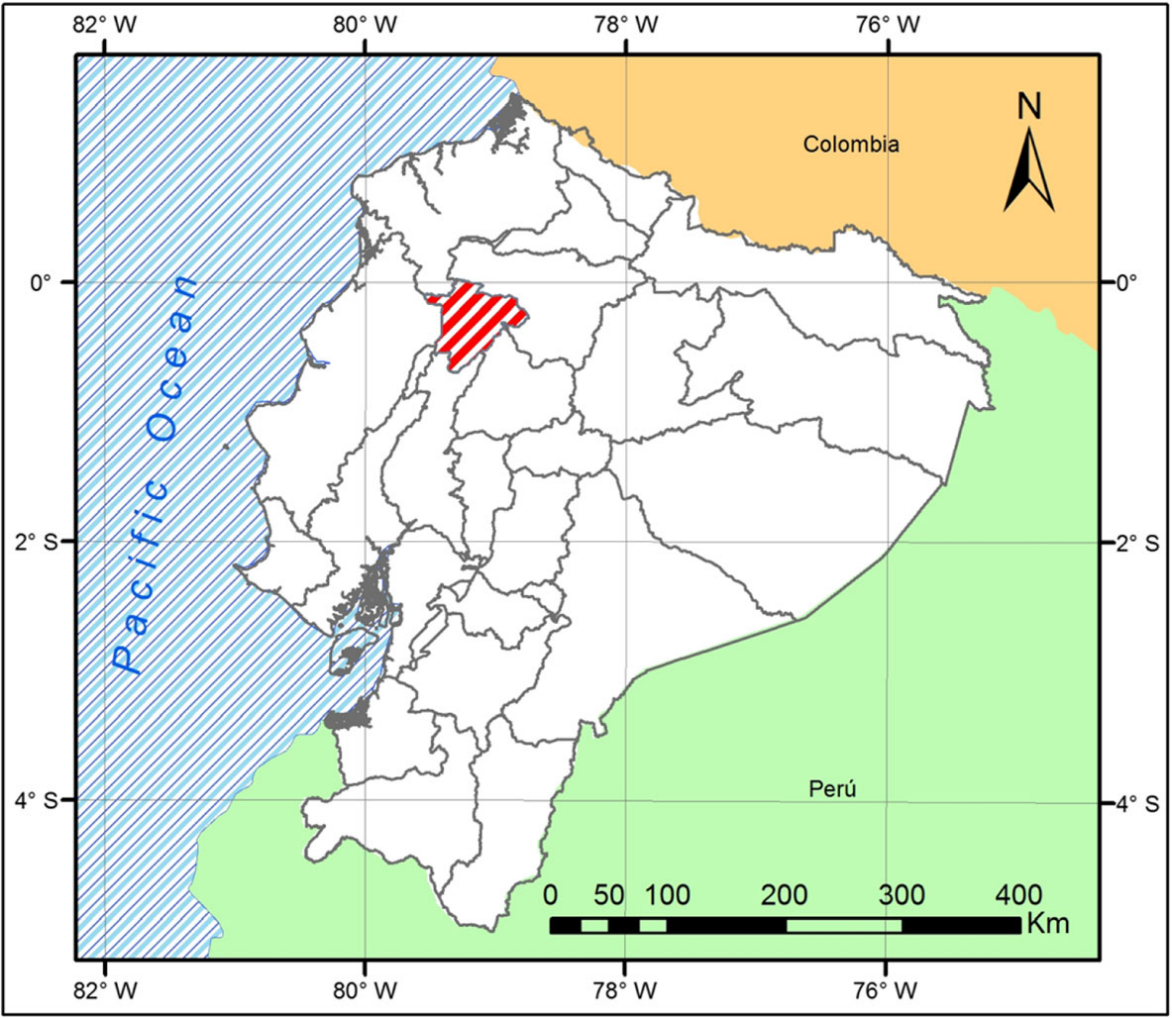
Location study area, province of Santo Domingo de los Tsáchilas in red lines

**Fig. 2:**
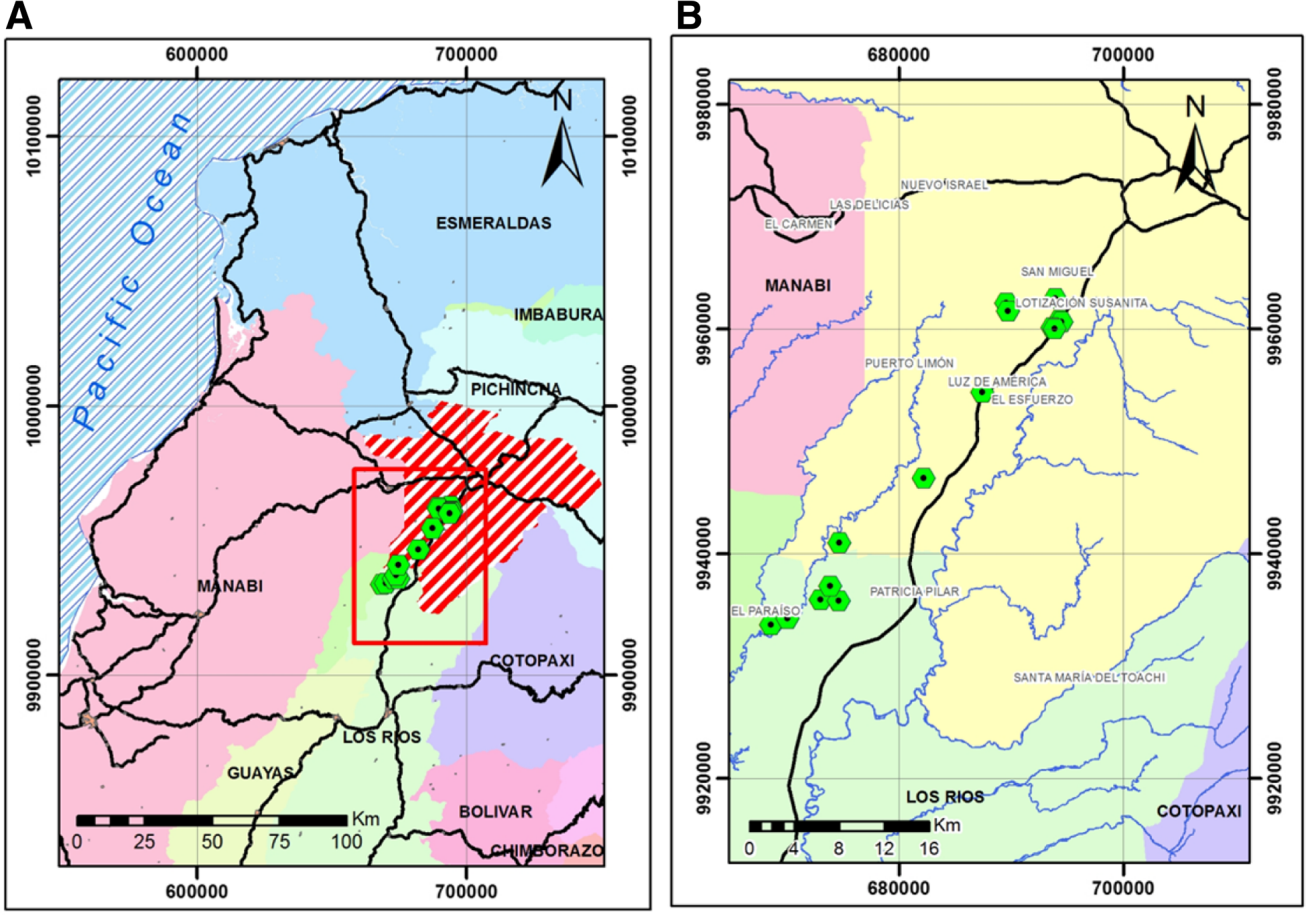
Sampling area in panel **a** and farm location in Panel **b**

In December 2014, a transversal study including two levels of sampling (herd and animal) was carried out. Blood samples were collected from cattle (*n* = 151) belonging to 15 dairy herds gathered in province of Santo Domingo de los Tsáchilas, humid tropical region at an altitude of 655 m above sea level [18] (Figs. 1 and 2).

### Collection of blood samples

Blood samples were collected at random, in 4 cm^3^-tubes with EDTA, through coccygeal vein puncture with 0.75 mm × 25 mm Vacutainer® needles. Samples were then stored at −20 °C until further use and analysis.

### DNA extraction

Genomic DNA was extracted from 1 mL of blood using a protocol previously described by J Sambrook and DW Russell [19]. Extracted DNA was eluted in 100 μl Tris-borate EDTA (TBE) and stored at −80 °C until further analysis. DNA concentration was determined using a NanoDrop ND-1000 (NanoDrop Technologies Inc., Wilmington, USA) b at 260 nm.

### PCR of msp5 and 16S rRNA genes

In order to perform the PCR allowing the detection of *msp5* gene, 100 ng of each sampled DNA were used. The PCR primer and technique used has been described previously [17, 20, 21] with some modifications. A total volume of 25 μL was composed of 2.5 PCR buffer, 0.5 U Platinum® Taq DNA Polymerase (Invitrogen™), 1 μM of each primer (Ana 19A/Ana 19B or RYaF16S/RYaR16S, Invitrogen™, as shown in Table 1 Invitrogen™), 0.2 mM of each nucleotide (dATP, dTTP, dCTP and dGTP; Invitrogen™) and 2.5 mM of MgCl_2_. The program used for *msp*5 gene in the thermal cycler (TC-512 TECHNE) consisted in: 5 min-incubation at 94 °C, followed by 35 cycles of 45 s at 94 °C, 30 s at 64 °C and 1 min at 72 °C. A final 10 min-extension at 72 °C ended the program. For 16S rRNA PCR, 68 °C was used for hybridization and the extension time was changed 1.5 min. PCR products were analyzed on 1.5% agarose gel in TBE (45 mM Tris-borate, 1 mM EDTA) and visualized with SYBER® Safe DNA in a UV-transilluminator.

**Table 1:** Sequence of primers used for amplification of *msp5* gene and 16S rRNA

Primer	5′-3′ Sequence	Temp. hybrid	Target	Reference and target
Ana 19A	GTGTTCCTGGGGTACTCCTA	64 °C	MSP5	Reyna-Bello et al., 1998
Ana 19B	TGATCTGGTCAGCCCCAGCT			
RYaF16S	TAACACATGCAAGTCGAACGG	68 °C		Chacón 2012
RYaR16S	ACCCAACCTTAAATGGTCGC			
M13 F M13 R	ACATTCCAGCAGCAGTTC GAG CACGTGAATCCTCAATTTTGT	63 °C	Fragment inserted into the plasmid	Invitrogen™

### Cloning

Two μL of each PCR sample (An-SD-1 and An-SD-18) were ligated to TOPO TA vector (TOPO TA Cloning*®* kit, Invitrogen™), following manufacturer’s instructions. Subsequently, electroporation was performed with the Gene Pulser Xcell™ (BIORAD™), according to the protocol described by the manufacturer, for the transformation of *E.coli* with ligated plasmid. Once the cells were electroporated, 1 mL SOC medium (composed of 2% Tryptone peptone, 0.5% yeast extract, 100 mM NaCl, 2.5 mM KCL, 100 mM MgCl_2_ and 100 mM MgSO_4_ at pH 7) was added and placed one hour for stirring at 225 rpm in a 15 mL-centrifuge tube placed horizontally. Later, 50 μL of cloning were spread on LBP Petri dishes (prepared according to Sambrook and Russell, [19]) containing 50 μg/mL Kanamycin [SIGMA™], 40 μL of 40 mg/mL X-Gal (Invitrogen™) and 40 μL 100 mM-IPTG (Invitrogen™); it was further stored 24 h at 37 °C.

### Purification of plasmid DNA of transformed dH5α *E. coli*

In all transformations of *E.coli*, 3 colonies were selected by colony PCR using the protocol described above using primers Ana 19A and B. These colonies were seeded in 10 mL LB culture medium [19] in the presence of 20 μg/mL Kanamycin (SIGMA™) for 24 h. Afterwards, cells were recovered through centrifugation of 3 mL from the culture at 10,000 xg for 10 min; plasmid DNA was further extracted from the pellet by a PureLink kit (K210010, Invitrogen™,USA).

### Sequencing

Extracted plasmid DNAs were sent to Macrogen™ sequencing company (Seoul, South Korea), in order to sequence the cloned fragments, using forward and reverse M13 universal primers.

### Sequence analysis

Cloning sequences were analyzed, cut and aligned in order to establish the consensus sequences with the MEGA6 Software; later, these consensus sequences were submitted to the BLAST database (NCBI Blastn) for identification and similarity.

### Statistical analyses

At first, the disease prevalence was estimated, both at farm and individual levels. A farm was categorized as positive if at least one PCR-*msp5* tested positive among all samples analyzed. On the other hand, the influence of gender as possible risk factor was assessed by estimating Odds Ratios (ORs). Statistical analyses were performed with Epi Info 7™ and R software.

## Results

Our transversal study was performed in December 2014; 151 bovine blood samples were collected in 15 dairy farms located in the commune Luz de América, province of Santo Domingo de los Tsáchilas, in Ecuador’s coastal region. The sample size reached 49.5% of all animals present in the farms (*N* = 305). No sampling bias was to be considered, as the maximal proportion reached 25.2% (*N* = 38) in SD-4 farm. Males represented 11.9% (*N* = 18) of animals sampled.

Prevalence reached 100% at herd level and 86.1% at individual level (130 positive animals). Herd prevalence ranged from 40.0% (2/5) in SD-7 farm, to 100% in farms SD-4 (38/38), SD-8 (14/14), SD-9 (3/3) and SD-13 (5/5). No significant difference was observed between males and females (*P* > 0.05; OR = 1.94 for females; CI = 0.57–6.62) when considering *msp5* PCR results (males = 77.0% and females = 87.2%). Table 2 summarises the distribution of samples and results of *msp5* PCR analysis.

**Table 2:** Summary of results obtained per farm by PCR for the detection of *A. marginale* msp5 gene

Parameter	Animals present in the farm (N)	Animals sampled			Positive PCRmsp5	
		N	% per farm	% sampled	N	%
Sex						
Male		18		11.9	14	77.8
Female		133		88.1	116	87.2
Farm						
SD-1	47	12	25.5	8.0	8	66.7
SD-2	15	6	40.0	4.0	5	83.3
SD-3	10	10	100.0	6.6	8	80.0
SD-4	38	38	100.0	25.2	38	100.0
SD-5	20	9	45.0	6.0	8	88.9
SD-6	7	7	100.0	4.6	5	71.4
SD-7	5	5	100.0	3.3	2	40.0
SD-8	20	14	70.0	9.3	14	100.0
SD-9	3	3	100.0	2.0	3	100.0
SD-10	3	3	100.0	2.0	2	66.7
SD-11	35	12	34.3	8.0	10	83.3
SD-12	14	4	28.6	2.7	3	75.0
SD-13	40	5	12.5	3.3	5	100.0
SD-14	20	12	60.0	8.0	10	83.3
SD-15	28	11	39.3	7.3	9	81.8
TOTAL	305	151	49.5	100.0	130	86.1

*N* = number

Out of all samples positive by *msp5* PCR (*N* = 130), two samples (An-SD-1 y An-SD-18) were selected in order to identify the rickettsia with 16S rRNAr PCR and further cloning and sequencing. Once consensus sequences were obtained for each clone, they were compared: sequences had 1383 bp and showed a 100% identity. After consulting Blast Database (NCBI Blastn), a 100% similarity was identified with GenBank (access number CP000030.1 ), registered for St Maries strain of *A. marginale* by Brayton and collaborators in 2005 [22].

## Discussion

Bovine anaplasmosis is a disease commonly reported on the five continents. The rickettsia is transmitted through biological and mechanical vectors, but also iatrogenically. The disease is considered as endemic in tropical and subtropical areas and responsible for economic losses in cattle herds [2, 7]. Nevertheless, no report of *A. marginale* in Ecuadorian cattle has ever been published in indexed journals to date. Only few studies were issued in local journals and dissertations.

Our transversal study aimed at assessing the prevalence of bovine anaplasmosis, both at herd and individual levels in a district of Ecuador coastal region. The presence of *A. marginale* was assessed by using a standardized PCR for *msp5* gene amplification, according to a procedure previously described [20].

All herds were identified as being positive, along with a high proportion of infected animals per herd from 40 to 100%. Our result confirms the fact that anaplasmosis is endemic in the area of study with 86,1%, as it is the case in other tropical and subtropical regions of Latin America [23]. A second PCR was developed to amplify the rickettsia 16S rRNAr gene in two samples positive by *msp5* PCR. After cloning and sequencing, the 1400 bp fragment showed a 100% homology with St. Maries strain of *A. marginale* [22]. Such result allow us to conclude that *A. marginale* is present in Ecuadorian cattle; the sequence we operated is commonly used to perform taxonomy among bacteria of the Order Rickettsiales, Family Anaplasmataceae [10, 24].

It is important to mention that no farm provided more than 50% of samples. In this study, there was a total of 151 animals, 18 (11.9%) were males while 133 (88.1%) were females due to the dairy characteristic of farms. However, sex was not identified as a risk factor, which confirms previous results [25].

Significant differences were highlighted between farms for what PCR/*msp5* results are concerned: 40% of results were positive in SD-7 farm vs. 100% in SD-4, SD-10 and SD-12 farms. It is not surprising, the longer animals remain in a farm, the higher the risk of transmission. The localization of farms has probably influenced the distribution of the disease. In the area of study, 86.1% of animals were positive for *A. marginale* without clinical signs (anaemia, fever or more than 1% *A. marginale* in smear) suggesting the circulation of *A. marginale* in apparently healthy animals or persistent infection [6]. And due to the high prevalence found, the concept of enzootic stability can also be used [4].

The PCR usually detects DNA traces from previous infections, however, in the case of bovine anaplasmosis this is not true because once *A. marginale* infects an animal, it becomes infected for years [6] allowing us to infer that a positive PCR animal is still infected with *A. marginale*.

On the other hand infection by blood parasites such as *Trypanosoma vivax*, *Babesia bovis* or *Babesia bigemina*, frequently identified in Latin America, causes a clinical picture quite similar, worthless from a diagnostic point of view (Reyna-Bello, 2014). PCR test is seen as an alternative for these cases, or for persistently infected animals [9, 20, 26]. The gene encoding *msp5* has been used in numerous PCR trials [20, 27], due to the fact that, as a single copy, it is highly conserved in all *A. marginale* isolates [9, 28].

In endemic areas, clinical cases are rarely observed, most often in naïve animals recently introduced (and coming from free areas). Animals persistently infected can be responsible for outbreaks in a naïve herd, when moved to a disease-free area where vectors are present [2]. The high prevalence of anaplasmosis estimated in the area of study and for previous reports of 85.5% in cattle sampled in Quevedo canton, province of Los Ríos [15] and 91.7% in cattle slaughtered in Quito Metropolitan Slaughterhouse [16], places the lowlands of Ecuador in enzootic stability for bovine anaplasmosis.

The moderate prevalence (68%) reported by Muñoz and collaborators in cattle of Zamora, Canton [14] is probably related to the lower sensitivity of the test used, i.e. blood smear, compared to PCR or ELISA [9]. Another study performed in the Galápagos Islands reported a 64.1% prevalence estimated by a commercial competitive ELISA (VMRD Inc.™) in a sample of 184 animals [29].

In neighbouring countries, anaplasmosis was reported to be present at prevalence rates similar to our study. In Colombia, for example, a 90.3% prevalence obtained by agglutination test was reported [30]; the performance of an ELISA using *msp5* recombinant protein as antigen revealed a prevalence ranging between 47% [17] and 94% [31, 32] in Venezuela. In Costa Rica, a prevalence ranging from 20.0 to 72.0% was estimated by using an ELISA/MSP5r kit (VMRD Inc.™) [25] while a prevalence of 15% was estimated in Texas, with the same kit [33]. Indeed, such differences in prevalence have been mentioned by other authors [1].

The distribution of the rickettsia depends on the presence of vectors, which are arthropods belonging to the Ixodidae family; the most important belong to the genera *Dermacentor* and *Rhipicephalus*. In Latin America, the tick most widely distributed is *Rhipicephalus microplus*, wich is known as vector of *A. marginale* [4]. Nevertheless, in Latin America, the epidemiological importance of ticks as vectors is controversial: the transmission by blood sucking dipterans, such as horseflies, would play a more important role [4, 5]. Distribution and prevalence of anaplasmosis are directly related with the epidemiological role played by the different vectors. *Dermacentor albipictus* was mentioned as the main vector of the disease in Texas [33]. In Latin America, the predominating tick is *Rhipicephalus (Boophilus) microplus* (*R. microplus*), which does not seem to be an efficient vector for *A. marginale* [4, 7]. Transmission of anaplasmosis was mentioned to be mainly operated by blood sucking dipterous, iatrogenic or vertical transmission in Latin America [7, 34]. Vertical transmission would depend on the type of strain involved but infecting until 25% of calves and sometimes causing their death [35, 36]. On the other hand, Scoles and collaborators (2008) reported that *Dermacentor andersoni* was more efficient in transmitting *A. marginale* than horseflies. Nevertheless, the relationship between pathogen and vector has not been studied widely in Latin America yet (Baldacchino et al., 2014). While it is true, *R. microplus* happens to be an efficient vector, but to be species-specific (one sole host) limits its vector capacity in Latin America and Africa, where it is the predominating species [23].

In this regard, differences between *A. marginale* strains have been described in the world; some strains of Florida and Mississippi are not transmitted by ticks [1].

A study carried out in Costa Rica pointed out the presence of horseflies as the major risk factor for bovine anaplasmosis, not the ticks presence [25]. It is possible that, like what happened when *T. vivax* ‘travelled’ from Africa to Latin America more than 100 years ago, it adapted from a transmission by Tse Tse fly in Africa to a horsefly transmission in America, after gene deletion in its kinetoplast [37]. *Anaplasma marginale* might also have adapted itself to vectors existing in Central and South America, which would explain such a high prevalence of the disease in the region.

## Conclusion

The high prevalence observed at farm and animal levels, as well as the molecular characterization of *A. marginale* in Ecuador, would allow to clarify the epidemiological situation of this hemotrophic, but also to better focus its diagnosis, treatment and control in Ecuador.

## Abbreviations

16S rRNA: 16S of ribosomal RNA
*A. marginale*: *Anaplasma marginale*
ELISA: Enzyme Linked Immunosorbent Assay
MSP5: Mayor Surface Protein 5
ORs: Odds Ratios
PCR: Polymerase Chain Reaction
*R. microplus*: *Rhipicephalus microplus*
